# Targeted plasma proteomics identifies a novel, robust association between cornulin and Swedish moist snuff

**DOI:** 10.1038/s41598-018-20794-3

**Published:** 2018-02-02

**Authors:** Anneli Sundkvist, Robin Myte, Stina Bodén, Stefan Enroth, Ulf Gyllensten, Sophia Harlid, Bethany van Guelpen

**Affiliations:** 1 Department of Radiation Sciences, Oncology, Umeå University, Umeå, Sweden; 2 Department of Immunology, Genetics, and Pathology, Biomedical Center, Science for Life Laboratory Uppsala University, Uppsala, Sweden

## Abstract

Lifestyle behaviors are believed to influence the body’s inflammatory state. Chronic low-grade inflammation contributes to the development of major non-communicable diseases such as diabetes, cardiovascular disease and cancer. Inflammation may thus be an important link between lifestyle and disease. We evaluated self-reported physical activity, tobacco use and alcohol consumption in relation to plasma levels of 160 validated inflammatory and cancer biomarkers. The study included 138 participants from a population-based cohort, all with repeated sampling of plasma and data ten years apart, allowing consideration of both intra- and inter-individual variation. Of 17 relationships identified, the strongest was an independent, positive association between cornulin (CRNN) and Swedish moist snuff (snus) use. We replicated the finding in a second cohort of 501 individuals, in which a dose-response relationship was also observed. Snus explained approximately one fifth of the variance in CRNN levels in both sample sets (18% and 23%). In conclusion, we identified a novel, independent, dose-dependent association between CRNN and snus use. Further study is warranted, to evaluate the performance of CRNN as a potential snus biomarker. The putative importance of lifestyle behaviors on a wide range of protein biomarkers illustrates the need for more personalized biomarker cut-offs.

## Introduction

Chronic low-grade inflammation is an established pathological mechanism in the development of major non-communicable diseases such as diabetes, cardiovascular disease, and cancer^1–4^. Inflammatory biomarkers, such as C-reactive protein (CRP), interleukin 6 (IL-6), and tumor necrosis factor alpha (TNF/TNF-α), originate from multiple sources such as adipocytes, endothelial cells, macrophages and skeletal muscle and are often altered in individuals suffering from these chronic diseases. Several lifestyle-related behaviors, including smoking, alcohol consumption, and lack of physical activity are also recognized as chronic disease risk factors and, at the same time, are believed to influence the inflammatory state in the body^3,5,6^. Inflammation may, therefore, be an important mediating step between lifestyle and disease.

The association between smoking, cardiovascular diseases and inflammation is well known, with higher levels of circulating inflammatory markers reported in smokers, as well as reduced levels after smoking cessation^5,7^. Similarly, the use of smokeless tobacco is associated with higher risk of myocardial infarction and stroke^8^. In contrast, the use of Swedish moist snuff (snus) does not seem to be associated with the risk of stroke or myocardial infarction^6,8,9^. A suggested explanation for the differences in morbidity risk between the use of snus and other smokeless tobacco is related to the manufacturing; Swedish snus is heat treated and not fermented, giving lower levels of carcinogenic tobacco-specific nitrosamines^10^.

Moderate alcohol consumption is reported to cause favorable changes in several cardiovascular biomarkers and to demonstrate inverse associations with inflammatory markers, including lower levels of CRP and IL-6^11,12^. In healthy individuals, free of cardiovascular disease, the dose-effect risk curve appears to be J-shaped, with excessive alcohol use leading to increased cardiovascular events and all-cause mortality^2^. The relationship with inflammation is not straight forward, with results of a meta-analysis showing no significant association between moderate alcohol intake and levels of CRP, IL-6, or TNF^13^. There is also convincing evidence that alcohol, even in moderate consumption (up to one drink per day, <12.5 g ethanol) increases the risk of cancer of the oropharynx, esophagus, liver, colon and breast^2,14^.

Regarding physical activity, results are conflicting.^15–18^. Although a transient pro-inflammatory state has been observed in the acute phase after strenuous exercise, longitudinal observational studies and intervention studies also support a long-term anti-inflammatory effect of regular physical activity^19,20^. However, a meta-analysis of randomized trials, failed to confirm an effect of aerobic exercise in reducing CRP levels^16^. Obesity also contributes to chronic, low-grade inflammation, and weight loss significantly increases IL-10 and decreases TNF-α and IL-6^21^, but there is limited evidence as to whether this anti-inflammatory effect is a result of the physical activity or a consequence of weight loss and changes in body composition^15–17,20^. Leisure time physical activity is reported to have less effect than body mass index (BMI) on the inflammatory markers IL-1β, IL-6. TNF, and CRP^22^.

Despite the central role of inflammatory processes in the development of several major non-communicable diseases, research to date has largely focused on a handful of biomarkers. For many novel inflammatory markers, very little is known about potential contributors to inter- and intra-individual variation, including lifestyle behaviors. And for established inflammatory markers, as summarized above, results have been conflicting. Both lifestyle factors and genetic factors have recently been found to have a strong impact on numerous circulating biomarker levels^23^. Panels of biomarkers, such as inflammatory and disease markers, have potential importance not only for understanding disease etiology, but also for use as risk-predictive, diagnostic, therapeutic response and prognostic markers, and as therapeutic targets. However, this requires a better understanding of how they are affected by parameters such as lifestyle behaviors.

Using a unique collection of repeated samples from the prospective, population-based Västerbotten Intervention Programme (VIP) cohort, we evaluated the impact of self-reported lifestyle behaviors in relation to a large panel of inflammatory and cancer biomarkers. The use of repeated samples allowed investigation of differences in biomarker levels both between and within participants over time.

## Results

### Participant characteristics

Characteristics of the participants at baseline and at the repeated sampling occasion are presented in Table 1. The median age was 50 years at baseline, and 60 years at the second sampling occasion. BMI increased modestly with time (0.7 kg/m^2^ difference in median BMI between baseline and repeat, p < 0.001). A small proportion of the participants quit smoking between baseline and the repeated sampling, yet there was no overall significant difference in smoking status between sampling occasions (p = 0.10). There were negligible changes in recreational physical activity, Swedish moist snuff (snus) usage, total alcohol intake, and unhealthy lifestyle score over time. Overall, recreational physical activity was low, with 43 and 46% reporting no physical activity at the first and second sampling occasion, respectively. Reported total alcohol intake was also low; median 3.5 and 4.0 grams/day at baseline and repeated sampling respectively, and only one participant reported a high-risk intake (>24 g ethanol/day for men and >12 g/day for women). For specific alcoholic beverages, beer consumption decreased over time (both 2.1% and 2.8–3.5% alcohol by volume (alc/vol), P = 0.001 and P < 0.001, respectively). Wine consumption increased over time (P < 0.001), while consumption of spirits did not change significantly over time (P = 0.33).

**Table 1:** Baseline and follow-up characteristics^a^.

Variable	Baseline	Repeat	P^b^
Baseline age, y			
Median	50	60	—
Age categories (N, (%))			
30 to <40	16 (12)	0 (0)	—
40 to <50	59 (44)	17 (13)	
50 to <60	58 (44)	61 (46)	
>60	0 (0)	54 (41)	
Sex			
Men	76 (57)	75 (57)	—
Women	57 (43)	57 (43)	
BMI, kg/m^2^			
Median	25.3	26.0	<0.001
BMI categories (N, (%))			
<25	63 (47)	53 (40)	
25 to <30	56 (42)	52 (39)	
>30	14 (11)	27 (20)	
Recreational physical activity (N, (%))			
Never	57 (43)	61 (46)	0.72
Every now and then—not regularly	35 (26)	35 (27)	
1–2 times/week	22 (17)	15 (11)	
2–3 times/week	13 (10)	12 (9)	
>3 times/week	6 (5)	9 (7)	
Smoking status (N, (%))			
Never smoker	52 (39)	57 (43)	0.10
Ex-smoker	42 (32)	51 (39)	
Current smoker	39 (29)	24 (18)	
Snus status (N, (%))			
Never user	91 (68)	89 (67)	0.85
Ex-user	13 (10)	11 (8)	
Current user	29 (22)	32 (24)	
Alcohol intake (grams/day)			
Median	3.5	4.0	0.34
High risk intake (>24 g/day for men, >12 g/day for women (N, (%))	1 (1)	1 (1)	1.00
Specific beverages (grams/day), median			
Beer 2.1% alc/vol	19.2	1.0	0.001
Beer 2.8–3.5% alc/vol	1.1	0.9	<0.001
Beer ≥4.5% alc/vol	1.5	1.2	0.50
Wine	0.8	20.0	<0.001
Spirits	0.3	0.3	0.33
Unhealthy lifestyle score^c^ (N, (%))			
0	50 (38)	48 (36)	0.98
1	60 (45)	61 (46)	
2	18 (14)	17 (13)	
3	5 (4)	6 (5)	
4	0 (0)	0 (0)	

^a^Five samples at baseline and six samples at repeat could not be analyzed for biomarkers due to technical problems. Consequently, those participants were excluded from further statistical analysis.

^b^Paired Wilcoxon signed rank test for continuous variables, chi-square tests for categorical variables.

^c^Score calculated by adding the number of met conditions of: BMI ≥ 30, smoking status = smoker, total alcohol intake >24 grams/day for men and >12 grams/day for women, or physical activity = “never”.

### Associations between biomarkers and lifestyle

**Figure 1:**
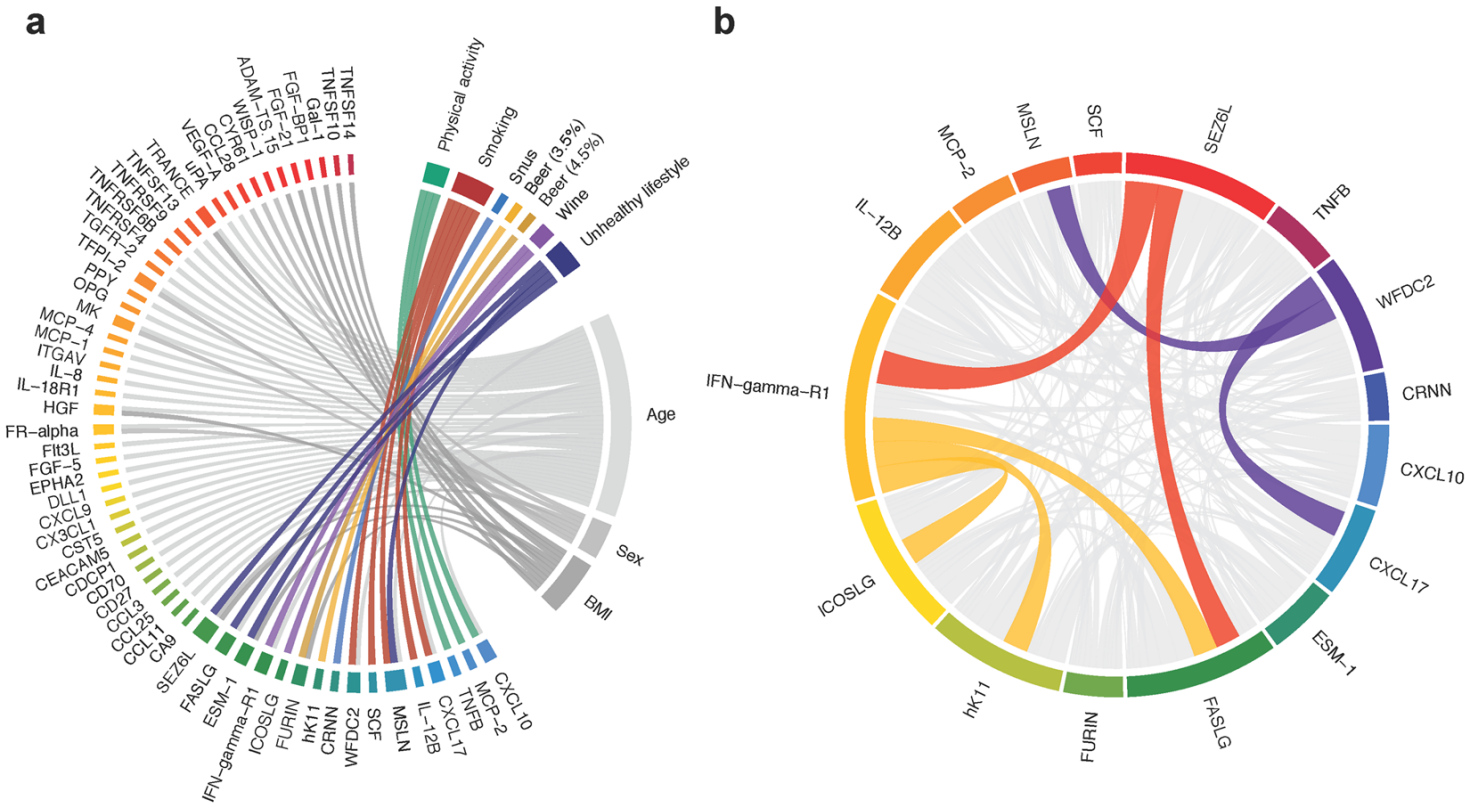
Protein biomarker associations. a) Circos plot of significant associations between proteins and lifestyle behaviors, shown in color. Connections illustrate significant contributions to protein variance (Bonferroni corrected P-value < 0.05). b) Partial correlations between the 16 proteins associated with lifestyle behavior, calculated by estimating Spearman’s correlations on the standardized residuals from mixed models adjusting for age, sex and lifestyle behaviors. Width of the links correspond to the squared correlation coefficient (R^2^). Colored links represent correlations with R^2^ >0.25. All correlations were positive.

Of the 160 proteins that passed quality control, 17 were significantly associated with seven lifestyle behaviors in the multivariable mixed models (Fig. 1a, Bonferroni-adjusted P < 0.05). Estimated parameters for significant associations are presented in Table 2. Estimates for all associations between proteins and lifestyle-behavior variables and other covariates can be found in Supplementary Data 1 online. Age was associated with 32 proteins (of which 30 were positive associations), sex with seven proteins (all higher in females), and BMI with 11 proteins. Similar analyses focusing on the metabolic syndrome, including BMI, are presented in a previous study^24^. None of the significant associations differed between CRC cases and controls (adjusted P-heterogeneity >0.05, Supplementary Data 2 online). Five other associations displayed significant heterogeneity between cases and controls, but since the associations were not statistically significant in the combined analysis adjusted for case-control status, they should be interpreted with caution.

**Table 2:** Statistically significant associations between plasma protein levels and lifestyle behaviors.

Variable	Protein	Estimate	Beta (SE)^a^	P^b^	R_m_^2^ (%)^c^
Physical activity (1–5, ranging from none to exercise ≥3 times a week)	TNFB^d^	Per unit increase	0.19 (0.04)	8.5*10^−6^	5
	MCP-2^d^	Per unit increase	0.17 (0.04)	9.1*10^−5^	4
	CXCL10^d^	Per unit increase	0.16 (0.04)	1.5*10^−4^	3
Smoking (vs. non-smokers)	CXCL17^e^	Smokers	0.75 (0.14)	5.2*10^−8^	10
	CXCL17^e^	Ex-smokers	0.12 (0.13)		
	SCF^d,e^	Smokers	−0.64 (0.16)	1.7*10^−4^	9
	SCF^d,d^	Ex-smokers	−0.20 (0.14)		
	MSLN^e^	Smokers	0.54 (0.13)	2.5*10^−9^	7
	MSLN^e^	Ex-smokers	−0.10 (0.12)		
	WFDC2^e^	Smokers	0.37 (0.15)	2.6*10^−4^	4
	WFDC2^e^	Ex-smokers	−0.12 (0.13)		
	IL-12B^d^	Smokers	−0.36 (0.14)	4.1*10^−5^	3
	IL-12B^d^	Ex-smokers	0.13 (0.12)		
Snus (vs. non-users)	CRNN^e^	User	1.22 (0.18)	8.5*10^−10^	23
	CRNN^e^	Ex-user	0.16 (0.29)		
Beer intake, 2.8–3.5% alc/vol (vs. zero intake)	hK11^e^	<Median intake	−0.55 (0.15)	2.3*10^−4^	2
	hK11^e^	≥Median intake	−0.75 (0.18)		
Beer intake, ≥4.5% alc/vol (vs. zero intake)	Furin^e^	<Median intake	0.08 (0.16)	2.2*10^−4^	2
	Furin^e^	≥Median intake	0.54 (0.18)		
Wine intake (vs. zero intake)	ICOSLG^e^	<Median intake	−0.74 (0.17)	1.4*10^−5^	7
	ICOSLG^e^	≥Median intake	−0.32 (0.21)		
	IFN-gamma-R1^e^	<Median intake	−0.47 (0.15)	9.3*10^−5^	4
	IFN-gamma-R1^e^	≥Median intake	−0.01 (0.18)		
Unhealthy lifestyle (0–4, score ranging from healthy to unhealthy)	ESM-1^e^	Per unit increase	−0.37 (0.07)	2.9*10^−7^	10
	FASLG^e^	Per unit increase	−0.27 (0.07)	8.8*10^−5^	5
	SEZ6L^e^	Per unit increase	−0.26 (0.07)	1.9*10^−4^	5
	MSLN^e^	Per unit increase	0.25 (0.06)	5.4*10^−5^	4

**SE**: standard error. **R**_**m**_^**2**^: variance explained by fixed factors.

^a^Regression coefficient from linear mixed models interpreted as standard deviation difference in protein levels compared to exposure reference group (categorical exposures) or per exposure unit increase in (for continuous exposures), adjusted for case status, age, sex, BMI, smoking, and total alcohol intake as fixed factors and participant and case set as random factors.

^b^Bonferroni-adjusted threshold: 0.05/160 = 3.125*10^−4^.

^c^Protein variance explained (R_m_^2^) by each lifestyle behavior variable. Calculated as the change in R_m_^2^ by adding the variable to a linear mixed model including case-status, age, and sex as fixed factors, and subject and case-set as random factors.

^d^Inflammation panel.

^e^Oncology II panel.

Three proteins (CXCL10, MCP-2, and TNFB) were positively associated with physical activity independent of BMI, although CXCL10 was also associated with age. Smoking was positively associated with levels of CXCL17, MSLN, and WFDC2, and inversely associated with levels of IL-12B and SCF. Snus was positively associated with cornulin (CRNN). Total alcohol intake was not significantly associated with any protein, but moderate intake of beer (2.8–3.5% and 4.5% alc/vol) was associated with slight changes in the levels of hK11 and furin, respectively. Moderate intake of wine was associated with lower ICOSLG and IFN-gamma-R1. A higher unhealthy lifestyle score (calculated by combining BMI status, physical activity, smoking, and alcohol habits) was associated with higher levels of MSLN and lower levels of ESM-1, FASLG, and SEZ6L. FASLG was also associated with age, while ESM-1 and SEZ6L were also associated with BMI, and MSLN was also associated with smoking.

Partial correlations between the lifestyle and behavior-associated proteins (adjusted for lifestyle behaviors, age, sex, and BMI) are presented in Fig. 1b. All correlations were positive. The strongest correlation was observed between SEZ6L and IFN-gamma-R1 (R^2^ = 0.40). IFN-gamma-R1 was also correlated with FASLG, hK11, and ICOSLG (R^2^ = 0.29 to 0.32). The three proteins that were positively correlated with smoking (WFDC2, CXCL17 and MSLN) were also correlated with each other (R^2^ = 0.27 to 0.31).

**Figure 2:**
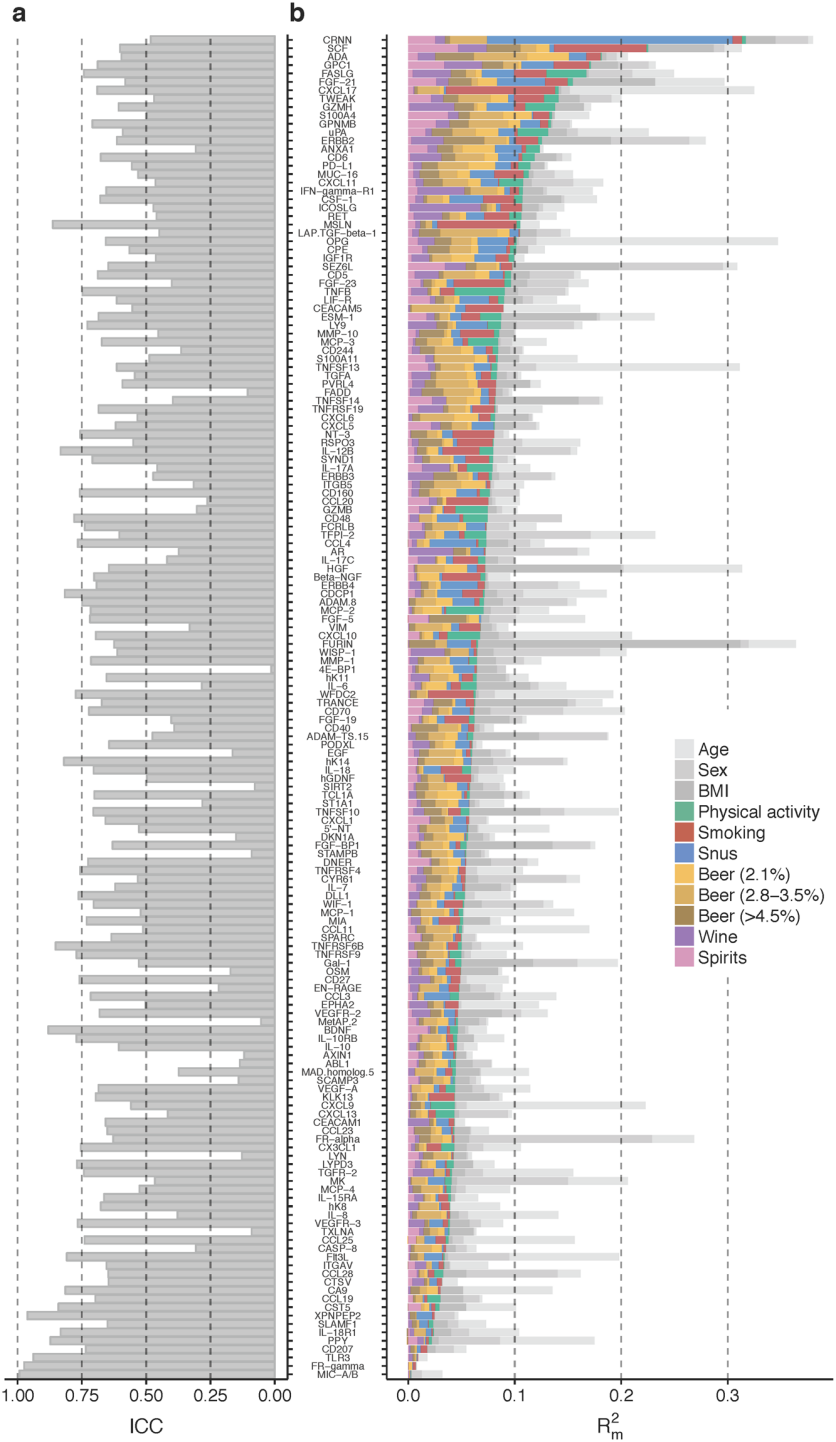
Protein biomarker variation. (**a**) Intraclass correlation coefficients (ICCs), i.e. proportion of inter-individual variance of total variance, for each protein calculated based on within- and between-individual variances estimated in linear mixed models including case-status, age, sex, BMI, and all lifestyle behavior variables as fixed factors, and subject and case-set as random factors. Proteins with high ICC vary less within, and more between participants, whereas proteins with low ICC vary less between and more within participants. (**b**) Protein variance explained (R_m_^2^) by each lifestyle behavior variable. Calculated as the change in R_m_^2^ by adding the variable to a linear mixed model including case-status, age, and sex as fixed factors, and subject and case-set as random factors.

Most of the proteins associated with lifestyle behavior displayed relatively low intra-individual variation over time with ICCs = 60 to 86%, see Fig. 2a and Supplementary Fig. 1. CRNN and ICOSLG displayed somewhat inferior ICCs compared to other proteins associated with lifestyle behavior (ICC = 48 and 47%, respectively). However, these low values were mainly driven by a few participants with large protein level fluctuations between sampling occasions (Supplementary Fig. 1). The approximate protein variance explained by each lifestyle behavior variable, as well as age, sex, and BMI, is presented in Fig. 2b. The combined proportion of protein variance (R_m_^2^) by lifestyle behavior, age, sex, and BMI varied between 1–31% for the 160 detectable proteins. Proteins BDNF, XPNPEP2, MIC-A/B, TLR3, and FR-gamma constituted a distinct group of proteins with low intra-individual variation (ICCs >80%) along with minor contributions from lifestyle variables (R_m_^2^ < 5%). Proteins AXIN1, OSM, 4E-BP1, SIRT2, STAMPB, TXLNA, EGF, SCAMP3, FADD, MetAP.2, DKN1A, on the other hand, displayed high intra-individual variation (ICCs <20%) but with equally low impact from lifestyle (R_m_ ^2^< 5%). The protein most influenced by any lifestyle behavior was CRNN which was significantly associated with snus (R_m_^2^ by snus: 23%), but not with any of the other lifestyle behaviors or background variables included in this study. Smoking explained 3–10% of the variation in the smoking-associated proteins, physical activity explained 3–5% in the physical activity associated proteins, and consumption of various alcoholic beverages explained 2–7% of the variance in proteins associated to alcoholic beverages. The unhealthy lifestyle score explained 4–10% of the variance in the proteins associated with that variable.

### Replication of the CRNN-snus association

The association between snus usage and CRNN was successfully replicated in the independent population-based Northern Sweden Population Health Study (NSPHS) (1.25 SD higher CRNN levels in current user vs. non-users, P < 2*10^−16^, R^2^ = 18%, Table 3), with an effect size very similar to that observed in the VIP (1.22 SD higher CRNN levels in current user vs. non-users, P = 8.5*10^−10^, R_m_^2^ = 23%). In the NSPHS, information on snus dose was also available (measured as the number of packs/week containing approximately 20 doses each, ranging from 0 to 14 packs/week). A larger snus consumption was linearly associated with increased CRNN (0.22 SD higher CRNN per 1 pack/week, P = 36*10^−15^, R^2^ = 12%).

**Table 3:** Replication of the CRNN-snus association in the independent NSPHS cohort.

	VIP (n = 133)			Replication NSPHS (n = 501)		
	n_baseline_/n_repeat_	Beta (SE)^a^	P	n	Beta (SE)^b^	P
**Snus usage**						
Non-user	91/89	ref		420	ref	
Current user	29/32	1.22 (0.18)	8.5*10^−10^	81	1.25 (0.12)	<2*10^−16^
Ex-user	13/11	0.14 (0.29)			**—**	**—**
		**R**_**m**_^**2**^ = 23%			**R**_**m**_^**2**^ = 18%	
**Snus dose**						
Per 1 pack/week		—	—		0.22 (0.03)	36*10^−15^
		—			**R**^**2**^ = 12%	

**VIP:** Västerbotten Intervention Programme**. NSPHS**: Northern Sweden Population Health Study. **SE**: standard error. **R**_**m**_^**2**^: variance explained by fixed factors. **R**^**2**^: variance explained.

^a^Regression coefficient from linear mixed models interpreted as standard deviation difference in CRNN to reference group (Non-snus users), adjusted for case status, age, sex, BMI, smoking, and total alcohol intake as fixed factors and participant and case set as random factors.

^b^Regression coefficient from linear regression interpreted as standard deviation difference in CRNN to reference group (Non-snus users) or change per 1 pack of snus/day, adjusted for age, sex, BMI, smoking, and total alcohol intake.

## Discussion

In this study, we evaluated the impact of modifiable lifestyle behaviors on plasma levels of 160 unique protein biomarkers related to inflammation and cancer. To determine intra-individual variability and time-dependent effects, we used repeated measurements taken 10 years apart. Our study identified 17 proteins associated with lifestyle behaviors, most of which displayed fair to excellent intra-individual correlation between measurements over time. Of the associations observed, the strongest was an independent positive association between CRNN and Swedish moist snuff (snus) use (Fig. 2). We were able to replicate this novel finding in a second cohort of 501 individuals, with snus explaining approximately one fifth of the variance in CRNN levels in both sample sets; 18% and 23%, respectively (Table 3). Furthermore, using the detailed tobacco data in the validation cohort, CRNN demonstrated a linear dose-response relationship with snus use.

The importance of the CRNN results are underscored by the fact that no snus-specific biomarkers have been reported in the scientific literature. CRNN was also the only protein associated with snus use in our analyses. Other factors, such as age, sex, smoking, BMI, and all lifestyle-behavior variables tested in our study, had little impact on CRNN levels. Nor did the association differ between CRC cases and controls. The sample size did not permit further subgrouping, by disease stage. However, the lack of heterogeneity between cases and controls and the similar magnitude of the relationship in the main and validation cohorts suggest that major differences by tumor stage are unlikely. The association between snus and CRNN may, therefore, prove to be a valuable tool in future research concerning the etiology of diseases potentially related to snus, in particular for distinguishing between health effects due to smoking as compared to other tobacco products, such as snus. Investigation of CRNN as a potential biomarker of snus use will require not only further validation in other populations, but also measurement of absolute CRNN concentrations to determine performance in terms of specificity and sensitivity and to establish meaningful cut-offs.

CRNN is an intra-cellular epithelial-induced stress protein primarily expressed in the upper layers of differentiated squamous tissues, including the esophagus and the epidermis, and it is a marker of late epidermal differentiation^25^. Loss of CRNN expression is associated with advanced tumor stage and poor survival in patients with esophageal squamous cell carcinoma^26^. According to the Public Health Agency of Sweden, approximately 18% of the men and 4% of the women in Sweden used snus daily in 2016. Thus, any potential use of CRNN as a disease marker might require personalized reference intervals based on snus use.

We identified two novel associations between intake of beer and the proteins furin and hK11. Furin was positively associated with beer (≥4.5% alc/vol) and BMI, while hK11 was negatively associated with beer (2.8–3.5% alc/vol) only (Fig. 2). Overexpression of furin has been correlated with increased aggressiveness in many types of cancers^27^. On the other hand, regarding hepatocellular carcinoma, overexpression of furin has a repressive effect on cancer cells^28^. hK11 (human kallikrein 11) is a protease suggested as prognostic and diagnostic biomarker of ovarian cancer and prostate cancer^29^. Although the variances of the levels of furin and hK11 explained by beer were low (R_m_^2^ ~3%), their specificity, when used as diagnostic and/or prognostic markers for diseases may be increased by introducing individual cut-offs for frequent beer drinkers and non-beer drinkers.

Smoking was positively associated with CXCL17, MSLN, and WFDC2, and negatively associated with IL-12B and SCF, in line with findings from other studies^23,30,31^. Smoking explained 9 and 5% of the variation in SCF and WFDC2, respectively (Table 2). WFDC2 (WAP four-disulfide core domain protein 2), also known as Human Epididymis protein (HE4) is a suggested biomarker for ovarian cancer^30^. However, systolic blood pressure has been reported to explain more of the variance (14.3%) of WFDC2 than smoking (1.7%)^23^, an association not observed in our data^24^. Moreover, renal failure is described as the most common cause of increased WFDC2 in patients with nonmalignant diseases^32^.

Physical activity was positively associated with three inflammatory biomarkers; CXCL10, MCP-2, and TNFB, of which CXCL10 was also associated with age. None of them was associated with BMI, which might make them suitable as biomarkers of physical activity. However, CXCL10 is reported to be increased in patients with cardiac heart failure and in patients with autoimmune diseases^33,34^. MCP-2 (Monocyte chemotactic protein 2) is a chemokine secreted during differentiation in muscle cells^35^. Regulation of muscle proliferation, differentiation, and regeneration is often proceeded by inflammatory processes and is thus dependent on pro-inflammatory stimuli. Previous studies imply that regular exercise of high intensity is required to achieve significant effects on biomarker levels^15^. The relatively low reported intensity and frequency of physical activity among the participants in the current study (Table 1), may explain the lack of associations between physical activity and the commonly studied inflammatory biomarkers IL-6, IL-10 and TNF. In addition, the relatively small sample size might limit us from detecting minor differences in biomarker levels.

Individuals with an unhealthy lifestyle (as calculated by our score, see Table 1) did not exhibit a specific protein profile, as the proteins associated with unhealthy lifestyle were more strongly associated with age, BMI, and smoking.

The use of biomarkers for risk-predictive, diagnostic, prognostic, or therapeutic purposes is complicated by the potentially large impact of lifestyle, and personal characteristics such as age, sex, and BMI. In addition, some proteins associated with lifestyle in the present study were also correlated with other proteins associated with the same, or other, lifestyle behaviors. Other factors such diseases, medications, and genetic factors can also influence biomarker levels, underscoring the potential of personalized biomarker cut-offs to improve both sensitivity and specificity^23^. Of all studied factors, age was the most commonly associated factor, associated with 32 biomarkers (Fig. 1a), followed by BMI, which we have previously described^24^. Similar results are published by Marques-Vidal *et al*. who concluded that age and BMI, followed by sex and smoking, significantly impacted inflammatory markers, while leisure-time physical activity showed little effect^22^. Being able to adjust for individual factors that interfere with the biomarker of interest thus provides an opportunity for more precise biomarkers of exposure, or biomarkers of risk prediction, early diagnosis and prognosis of non-communicable diseases.

The inflammatory state of the body is a dynamic equilibrium and can undergo dramatic changes, in infection or auto-inflammatory disease, for example. Confounding of associations between inflammatory biomarkers and lifestyle behaviors could certainly be possible, by more frequent infections in smokers compared to non-smokers, for example. However, although we could not account for acute or higher-grade inflammation, any major effect on our results seems unlikely, and the intra-individual variation between samples collected 10 years apart was generally low in the present study.

One major limitation of this study is that potential inducers to variation in biomarkers, such as genetic variation, diet, diseases, and medication were not included in this study. Due to technical limitations, CRP, a well-known biomarker of inflammation, was not included in the Olink inflammation panel. TNF-alpha, another well-known inflammatory biomarker, was excluded due to missing values. In the questionnaire, recreational physical activity referred to planned exercise in training clothes, and hence, did not include other activities such as walks in ordinary clothes, biking to work, occupational activity, or household activity. Furthermore, self-reported data on physical activity, tobacco use and alcohol consumption involves a risk of response bias and misclassification due to over- or under reporting by the participants^36^. However, although self-reported smoking data tend to be somewhat underreported, the correlation between self-reported smoking and actual prevalence of smoking is generally good^37^. Underreporting of use in this study would be unlikely to explain the association identified with CRNN. On the contrary, the association may actually be even stronger.

A major strength of this study is the analysis of repeated samples collected ten years apart, giving the opportunity to evaluate the proportion of intra-individual variation and time-dependent differences. The participants were from a population–based cohort. All blood samples were acquired after at least 8 hours of fasting, were frozen within an hour of collection, and had not been previously thawed. These strict criteria contribute to the validity of the study. The large number of biomarkers evaluated in relation to lifestyle behavior greatly contributes to the importance of this study. Finally, the fact that we were able to replicate the association between snus and CRNN in an independent sample set further increases the validity of this study.

Our main finding was a novel, robust association between circulating levels of cornulin (CRNN) and use of snus. The association was unrelated to smoking behavior and replicated in an independent sample set, in which it also demonstrated a dose-response relationship. In addition, CRNN was not significantly associated with any of the other background or lifestyle-behavior variables tested. Thus, further study is warranted, to evaluate the performance of CRNN as a potential novel biomarker for snus use. Among the other lifestyle behaviors studied, smoking stood out as the most influential factor, affecting the levels of five out of the 17 significant biomarkers. In conclusion, lifestyle behaviors may have significant impact on biomarkers and should be considered and adjusted for, if possible by using personalized cut-offs.

## Materials and Methods

### Study population and sampling

This investigation is based on a nested case-control study of colorectal cancer (CRC), in which all study participants were selected from the Västerbotten Intervention Programme (VIP), initiated in 1985 and still ongoing^38^. All residents of the county are invited to a general health exam at ten year intervals, starting at 40 years (at 30 in 1990–1996). Participants also donate a blood sample and fill out an extensive questionnaire on health, medical history, socioeconomic conditions, and lifestyle factors (e.g. smoking, alcohol and dietary habits).

Participants for this study were originally selected as part of a prospective study of biomarkers of CRC, with samples collected between August 28, 1990 and May 08, 2013. All CRC cases had to have a verified CRC diagnosis within five years after the latest sampling (excluding samples collected within three months of diagnosis) and to have at least two VIP blood samples. Blood samples were collected in EDTA vacutainers, centrifuged at 15 min, 1500 g (3000 rpm), separated into fractions of plasma, buffy coat, and erythrocytes and frozen within one hour. The fractions used in this study were stored at −80 °C until aliquoted for analysis (i.e. no previous freeze-thaw cycles). All case sets had samples collected ten years apart, with the exception of one with 20 years. We selected an equal number of control subjects, matched on age (+/− 12 months), sex, and sampling date (+/− 12 months). Controls had to be cancer free at the latest follow up (Dec. 31, 2014). For both cases and controls, only samples collected after at least eight hours of fasting were included. The final study included repeated samples from 69 prospective CRC cases and 69 matched controls, resulting in a total of 276 samples.

The project was approved by the Regional ethical review board at Umeå University, Sweden (Dnr 2015/172-32). All VIP participants provide a written informed consent donating their samples for research purposes, and they retain the right to withdraw that consent at any time in the future. All experiments in the study were performed in accordance with relevant guidelines and regulations.

### Protein biomarkers

All 276 samples were analyzed simultaneously for 178 unique protein biomarkers on two pre-designed Proseek Multiplex® immunoassay panels; Inflammation panel and Oncology II panel (Olink Proteomics, Uppsala, Sweden). The entire list of 178 proteins can be found in Supplementary Table S1. Plasma was analyzed using Proximity Extension Assay (PEA), which has high specificity and sensitivity^39^. Processing, output data quality check and normalization were performed by Olink Proteomics. Eleven of the samples, five from the baseline sampling and six from the repeated sampling, could not be analyzed due to technical problems. These included both baseline and repeated samples for two participants. Participants linked to those samples were thus excluded in further statistical analyzes. All data were delivered as Normalized Protein eXpression (NPX) values on a log2 scale. The log2 NPX values were scaled to mean 0 and SD 1 before data analysis, to facilitate comparisons between protein associations. Validation data and limits of detection (LOD) are available at the manufacturer’s webpage (http://www.olink.com). Data values below LOD were removed from the dataset. Proteins with >50% missing values; ARTN, IFN-gamma, IL-1-alpha, IL-2, IL-2RB, IL-4, IL-5, IL-10RA, IL-13, IL-20, IL-20RA, IL-22.RA1, IL-33, IL-24, LIF, NRTN, TNF and TSLP, were also excluded, leaving 160 proteins for the downstream data analysis.

### Replication of the CRNN-snus association

We investigated the CRNN-snus association in the independent Northern Sweden Population Health Study (NSPHS). The cohort has been described in detail elsewhere^23^. In short, the NSPHS was initiated in 2006 and consists of individuals participating in a health survey in the parish of Karesuando in Norrbotten County, Sweden. Each participant donated a blood sample, stored at −70 °C on site, and filled out an extensive questionnaire on medications and lifestyle (including snus usage). Plasma was analyzed for CRNN using the same PEA technique as for the VIP samples, as described above (Olink Proteomics, Uppsala, Sweden).

### Statistics

All computations were conducted in R v.3.3.2 (R Foundation for Statistical Computing, Vienna, Austria). Associations between protein markers and lifestyle were determined by fitting linear mixed models for each protein using the lmer function in the ime4 R-package. The mixed models included participant and the matched case-set as random factors (random intercept), all other variables as fixed factors. Variables included were CRC status (case, control), age (continuous), sex (male, female), BMI (continuous, z-transformed within sexes), recreational physical activity (5-level scale from never to >3 times/week), smoking (never-, ex-, current smoker), Swedish moist snuff (snus) use (never-, ex-, current user), intake of alcohol (grams/day) estimated from food frequency questionnaires (beer, analyzed separately by beer types sold in Sweden, with 2.1%, 2.8–3.5%, or ≥4.5% alc/vol, wine, spirits, as well as total alcohol intake). Total alcohol intake was analyzed as a continuous variable, while intakes of specific beverages were analyzed as categorical variables because of skewed distributions (three groups: zero intake, above/below sex-specific medians in non-zero participants to represent moderate/above-average alcohol consumption). Due to limited medication data from the questionnaire, medication use was not considered in the analyses. We also measured unhealthy lifestyle by calculating a score ranging from 0–4, in which one point was added for each of the following conditions: BMI ≥ 30, smoking status = “current smoker”, total alcohol intake >24 grams/day for men and >12 grams/day for women, and physical activity = “never”.

The contribution of each lifestyle variable to protein variance was tested by an analysis of variance approach using the anova.lme function with Satterthwaite approximation for degrees of freedom. For evaluation of variation in protein levels within and between individuals, we calculated intraclass correlations (ICC), defined as the proportion of total variance due to variation between individuals, using the variance estimates from the mixed models. We also calculated variance explained by fixed factors (R_m_^2^) using the RsqGLMM function in the MuMin package. Model assumptions were evaluated by visually inspecting Pearson standardized residuals. Outliers, defined as standardized residuals >3, were excluded separately for each protein in the mixed models (53 protein measurements in total, at most 2 per protein). In the replication study we had data on snus dose and the association between z-transformed CRNN concentrations and snus was assessed using linear regression (n = 501).

Missing values for the lifestyle variables were set to the value from the other sampling occasion. Missing values for the protein biomarkers were excluded separately in each analysis (complete case analysis). We assessed lifestyle-adjusted associations between significant proteins by calculating partial Spearman’s correlations on the estimated residuals from the mixed models using the cor function on pairwise complete observations. All P-values were adjusted for multiple testing using the Bonferroni method. Adjusted P-values < 0.05 were considered significant.

## Electronic supplementary material

Supplemental material

Supplementary Dataset 1

Supplementary Dataset 2
